# Care Home and Home Care Staff’s Learning during the COVID-19 Pandemic and Beliefs about Subsequent Changes in the Future: A Survey Study in Sweden, Italy, Germany and the United Kingdom

**DOI:** 10.3390/healthcare10020306

**Published:** 2022-02-05

**Authors:** Agneta Malmgren Fänge, Jonas Christensen, Tamara Backhouse, Andrea Kenkmann, Anne Killett, Oliver Fisher, Carlos Chiatti, Connie Lethin

**Affiliations:** 1 Department of Health Sciences, Lund University, 221 00 Lund, Sweden; agneta.malmgren_fange@med.lu.se (A.M.F.); connie.lethin@med.lu.se (C.L.); 2 Department of Social Work, Faculty of Health and Society, Malmö University, 205 06 Malmö, Sweden; 3 School of Health Sciences, University of East Anglia, Norwich NR4 7TJ, UK; tamara.backhouse@uea.ac.uk (T.B.); a.killett@uea.ac.uk (A.K.); 4 Center for Aging, Catholic University of Applied Sciences Munich, 836 71 Benediktbeuern, Germany; andrea.kenkmann@ksh-m.de; 5 Department of Economics and Social Sciences, Università Politecnica delle Marche, 60121 Ancona, Italy; o.fisher@inrca.it; 6 Centre for Socio-Economic Research on Ageing, IRCCS INRCA—National Institute of Health and Science on Ageing, 60124 Ancona, Italy; 7 Tech4Care srl, 60015 Falconara Marittima, Italy; c.chiatti@tech4care.it

**Keywords:** care home, COVID-19, digital technology, home care, learning, organization, resilience, staff experience, survey

## Abstract

The aim of this study was to compare perceptions of learning from the COVID-19 pandemic and beliefs in subsequent changes for the future, among care home and home care staff, in four European countries. A 29-item on-line questionnaire was designed in English and later translated into Swedish, Italian, and German on the impact of the pandemic on stress and anxiety. Anonymous data from care staff respondents was collected in four countries between 7 October 2020 and 17 December 2010: Sweden (*n* = 212), Italy (*n* = 103), Germany (*n* = 120), and the United Kingdom (*n* = 167). While care staff in all countries reported learning in multiple areas of care practice, Italy reported the highest levels of learning and the most agreement that changes will occur in the future due to the pandemic. Conversely, care staff in Germany reported low levels of learning and reported the least agreement for change in the future. While the pandemic has strained care home and home care staff practices, our study indicates that much learning of new skills and knowledge has taken place within the workforce. Our study has demonstrated the potential of cross-border collaborations and experiences for enhancing knowledge acquisition in relation to societal challenges and needs. The results could be built upon to improve future health care and care service practices.

## 1. Introduction

The COVID-19 pandemic has excessively affected older people and staff in long-term care [1]. During the early stages of the pandemic, front-line care staff were suddenly placed into a state of uncertainty and had many questions and concerns about how to cope with the unfolding new care context [2,3]. Many older people receiving long-term care, and social care workers, died from COVID-19 as it spread through care homes [1,4,5]. Consequently, new practices were needed for care staff were rapidly required to control and manage the pandemic. These included the increased and changed use of personal protective equipment (PPE), which was initially under resourced in care homes [6], augmented infection control practices [7], digital technology (DT) [8,9], and communication strategies [8,10]. Most importantly, even though treatment procedures for symptoms similar to COVID-19, to a large extent, were used initially [11], new knowledge had to be developed during the crisis, with little or no time for reflection [12].

In parallel, the crisis faced by the society at large and the care sector, caused by the COVID-19 pandemic, has called for the implementation of crisis management strategies on individual and organizational levels [13]. Crises are characterized by short decision times and needs for change [14], as tension, stress, and uncertainty affect the perceptual, affective, and motivational dimensions of decision-making [15]. Crises can trigger learning and development of new knowledge and skills both on individual and organizational levels [16]. Most importantly, considering the detrimental consequences from the COVID-19 pandemic for long-term care, it is vital that learning and new knowledge are taken forward to prevent unnecessary deaths and long-term health consequences in the future [17], as well as to improve resilience capacity among care staff. Resilience includes the capacity to adapt to new circumstances and make anticipations about a positive future [18]. Individual resilience plays a significant role in decision-making processes, disaster preparedness [19], and performance in stressful situations [20,21]. Research has also demonstrated the positive impact of educational interventions on perceived knowledge and resilience [17].

The use of digital technology (DT, e.g., electronic tools, automatic systems, technological devices, and resources that generate, process, or store information) in care and services has been expanding over recent years. DT has been identified as a key driver for increasing organizations’ efficiency and effectiveness [22]. Despite its potential, however, DT remains an untapped resource in most organizations [23], and the uptake is slow [24]. The importance of supporting the professionals achieve the required skills and knowledge to exploit them efficiently has been highlighted [25]. Each country reacted to the pandemic independently of other countries. Some countries that suffered from the pandemic earlier tried to find out which response measures worked, or which did not, given not only the epidemiological but also the economic and social aspects [26,27]. Before the COVID-19 pandemic, there were differences across countries in the use of DT for both private matters and for use in care services [28]. The pandemic has caused a rapid shift towards the remote delivery of care through online technologies [29], and DT has been put forward as an important tool to advance care and services [30].

To sum up, the COVID-19 pandemic has called for health and care professionals to develop new knowledge and skills and to adapt to a changing care context, with limited possibilities to anticipate future demands on them. Differences between countries were seen in the use of different measures within care and services, but whether such differences also include learning and prospects about the future remains to be investigated. Therefore, this study aims to investigate differences between countries in learning new knowledge and skills during the COVID-19 pandemic, among care and home care staff, and differences between countries in the beliefs about care practice and perceptions about change in the future due to the pandemic. It also aims at investigating the associations between new knowledge, learning, and beliefs about the future across countries during the COVID-19 pandemic.

## 2. Materials and Methods

### 2.1. Study Design

This study applied an exploratory, cross-sectional design, using an online survey in four European countries: Sweden, Italy, Germany, and the United Kingdom (UK).

### 2.2. Data Collection

#### 2.2.1. Questionnaire

A 29-item questionnaire was designed. The questionnaire was created in English and later translated into Swedish, Italian, and German. All variables were carefully duplicated to make sure the wording worked in each national context. The questionnaire contained items related to age, gender, role in the organization, and type and location of organization the participants worked in. In Italy, Germany and the UK regions were obtained, and in the German and UK questionnaire there was also distinction between home care and care home staff. Questions on the impact of the pandemic on stress and anxiety level were measured on a five-point scale, ranging from 1 = no impact to 5 = very strong impact. Following this, the participants were asked 22 questions to indicate their level of agreement. The questions covered four categories: “Support from the organization”, “External support for the organization”, “Learning experiences”, and “Outlook for the future”. A scale (five-point), starting with 1 = strongly agree ending with 5 = strongly disagree, was used (see Supplementary Material). This paper presents data on “Learning experiences” and “Outlook for the future”, while data on the two other categories have been presented in Lethin et al. [31].

#### 2.2.2. Procedure

Before the questionnaire was disseminated, usability and technical functionality of the on-line questionnaire was tested. Convenience and snowball sampling methods were applied, with authors disseminating the link to the one-page open survey through social media (LinkedIn, Twitter, and Facebook, including relevant care staff groups on Facebook), authors’ professional networks, and additional relevant channels such as newsletters for care workers. At the beginning of the questionnaire information about study purpose, data management, analysis, dissemination, and details for lead researchers were provided to respondents. By filling in the questionnaire the respondents concurrently gave their informed consent to participation. The survey had no timeline and was live between the 07.10.2020 and the 17.12.2020. All respondents were able to review and change their answers before submitting the survey. No incentives were offered to respondents. The survey was voluntary, and all responses were anonymous.

Study data were collected and managed using REDCap tools hosted at Lund University, Faculty of Medicine, Lund, Sweden. REDCap is a secure, web-based software platform designed to support data capture for research studies, providing (1) an intuitive interface for validated data capture; (2) audit trails for tracking data manipulation and export procedures; (3) automated export procedures for seamless data downloads to common statistical packages; (4) procedures for data integration and interoperability with external sources [32,33].

#### 2.2.3. Participants

In total, 602 participants participated and were included in this study. Mean age was 43 years, highest in Sweden (46 years) and lowest in Germany (39 years), of which the majority were female. The distribution of the participants across countries and professional roles was scattered, with most of the registered nurses (59%) being German and the majority of the nurse assistants (57%) being Swedish. The sample from the UK contained the majority (39%) of participants identifying as managers or as other care staff. For more detailed information about participant characteristics, see Lethin et al. [31].

### 2.3. Data Treatment and Analysis

Before data analysis, data were transformed into 1 = strongly disagree and 5 = strongly agree. Thereafter, data were analyzed at question level, and calculated for each profession and country, in total and for differences between the countries. ANOVA tests were used for calculating differences between the countries. Regression analyses were performed to investigate independent factors associated with the belief that, in the future, digital technology will be more common in care services, and with the belief that, in the future, there will be a stronger collaboration between professionals across organizations. Following the results of the bivariate analysis, conceivable explanatory factors were tested in the model whether they had a *p*-value ≤ 0.25. Consequently, two regression models related to the independent factors, associated with the belief that in the future DT will be more common in care and services, have been included. The first model only includes study sample characteristics. The second model also includes two variables related to learning new knowledge and skills during the COVID-19 pandemic, as these were found to be significant predictors of the belief that, in the future, DT will be more common in care services. Predictor variables was controlled by testing each independent variable against another through linear regression analysis. The data analysis was done in SPSS 27.0 and STATA 14, so *p*-values ≤ 0.05 were deemed to be significant.

## 3. Results

### 3.1. Learning of New Knowledge and Skills

Across all countries, the group of managers/coordinators learned most compared to other staff groups. Total means show that German staff reported the least new knowledge and skills across all knowledge categories, as did registered nurses across all countries. Italian staff professed the most agreement that they had learned new knowledge and skills across all categories, except in relation to infection control, where Sweden and the UK show slightly higher agreement. As shown in Table 1, Italian staff, to a larger extent, seemed to have learned new knowledge and skills related to crisis management compared to staff in the other countries.

As for clinical and care standards, the greatest agreement between countries was reported for new knowledge and skills in usage of PPE, with staff in Sweden, Italy, and the UK having most agreement to learning in this area. Conversely, staff in Germany reported most agreement for learning new knowledge and skills in infection control. However, this agreement was lower than that of all other countries (Table 2).

When it comes to learning new knowledge and skills related to the use of DT, the level of learning was lower compared to crisis management and clinical and care standards, respectively. Across countries, it seems that the level of learning, in general, was higher in Sweden, at least for managers/coordinators and registered nurses, and for most purposes of DT use. Nursing assistants in Italy reported that they acquired new knowledge and skills on how to use DT for different purposes compared to nursing assistants in the other countries. Across countries and staff categories, new knowledge about DT use, to the largest extent, was learned for communication purposes (Table 3).

### 3.2. Beliefs about the Future

As shown in Table 4, there was high agreement, across all four countries, that DT would be used more in the future and, across Sweden, Italy, and the UK, that clinical protocols for infection control will become more common. Overall, respondents neither agreed nor disagreed that it would be easier to attract new colleagues in the future. However, German respondents mostly disagreed, and the majority of Italian respondents agreed. In Sweden, Italy, and the UK, there was general agreement that the future may hold stronger collaborations between professionals across organizations, with Italian nursing assistants strongly agreeing. However, all staff groups in Germany, as well as Italian registered nurses, neither agreed nor disagreed.

Overall, there was low-level agreement across countries that the public image of care staff will be better in the media, with Swedish managers/co-ordinators agreeing more strongly. Staff from Germany neither agreed nor disagreed. Across all categories, Italian staff agreed most that changes would occur in the future. German staff reported the least agreement that there would be changes in the future across all categories, as did registered nurses across all countries.

### 3.3. Associations between New Knowledge and Learning and Beliefs about the Future

The association between beliefs of future use of DT, in care and services, and learning new knowledge and skills related to crisis management is relatively weak. A strong common belief that learning new knowledge and skills related to the use of DT will be more common to support care and services can be seen. Registered nurses are more willing to express their view versus nursing assistants and others. Using Germany as a reference country, the strongest and most outspoken beliefs can be seen in Italy (Table 5).

## 4. Discussion

This study investigated care home and home care staff’s learning of new knowledge and skills during the pandemic, as well their beliefs for subsequent changes in the future. The results showed that even though a crisis such as the COVID-19 pandemic may be disruptive for individuals, care and service organizations, and society, it seems as though care staff in all countries learned new knowledge and skills across all, or nearly all, practice areas to a quite significant extent. This outcome is one positive aspect to emerge from the pandemic and shows that, in times of great strain and difficulty, practices can still change.

One of the findings of this study was that, in all four countries, the managers/coordinators increased their knowledge and skills to a larger extent compared to other professional groups. Staff working directly with clients are trained to apply evidence-based protocols developed for different situations and diseases in their practice [34]. This includes using the best available evidence for all interventions, which, in the case of COVID-19, initially meant to use evidence related to similar diseases and symptoms [35,36]. Thus, instead of learning new knowledge and skills, care professionals might have utilized previous knowledge and skills in the care to a larger extent than staff in management or coordination positions. To some extent, this is supported by findings from Italy during the first wave in early spring 2020, where care professionals despite the challenges managed to cope with the situation relying on previous knowledge and skills [37,38,39,40]. Despite not being directly responsible for the clients’ care, from the managers/coordinators perspective, they faced many challenges and demands in their professional roles and tasks, i.e., the demands for crisis management skills were most prominent in this staff category.

Crisis management requires decisive, visible, and communicative leadership [41] with a focus on collaboration, coordination, and support [42]. The COVID-19 pandemic urgently called for new ways of communication and collaboration in care services, due to, e.g., isolation requirements [37,43], and it was, most probably, the task for the managers/coordinators to take the lead. In-depth knowledge about the practice and organization you are leading in times of crisis increases the possibilities that the infrastructure is relevant to the needs [44,45] and supports the implementation of evidence-based care strategies [34,46,47]. In this context, it is relevant to note that across countries and staff categories new knowledge about DT use, to the largest extent, was learned for communication purposes. Even if country differences were found in relation to DT use, with staff in Germany demonstrating the least learning among all staff categories, it is reasonable to assume that the physical distance requirement made all staff focus on developing digital communication routines. Moreover, in Germany, for example, care home staffing is hierarchical, where managers feel supported, but other staff groups may not [31], which may indicate that a supportive environment is required for learning to take place [48].

Health care is likely to become increasingly digital, and there will, most probably, be a need for the alignment of international strategies for the regulation, evaluation, and use of DT to strengthen pandemic management, and future preparedness for COVID-19 [49]. A UK report [50], stated the potential of DT to transform the health and social care system has still not been realized, though the COVID-19 pandemic has caused a rapid shift towards the remote delivery of care through online technologies. For the health and social care sector to make the most of emerging technologies, there need to be fundamental changes in how new tools are evaluated and supported during implementation.

As for learning related to clinical and care standards (PPE and infection control), here, the level of learning was consistently lower in Germany compared to other countries, independent of staff category. The reason might be an already high level of knowledge in these areas, leaving less room for learning. Research implies that rapid training of health professionals in treatment and training protocols may be a source of concern due to risks to the quality of care, as well as to the protection and safety of patients and health care providers [51]. The fact that most learning occurred in relation to clinical and care standards could also have consequences for care models, with augmentation in these areas contributing to further medicalization of care. That is, other important aspects of care were at risk of being put aside during the first phase of the pandemic [37,52]. However, it is evident that the situation in care homes and home care services, during the second COVID-19 wave in the fall 2020, called for rapid interventions related to infection control. It is likely that care staff had to quickly learn new knowledge and adopt new skills to keep pace with multiple new risks to staff and residents [53]. Changes in practice in response to new evidence are quite slow, and this is often referred to as a theory-practice gap [54]. Implementing new knowledge and methods into practice is a complex process with many facets, requiring combined effort of stakeholders in interaction, and teamwork is a necessary condition for the knowledge translation process [55]. Thus, adapting staffs’ skills and roles to the post-pandemic ways of working will, probably, be crucial to develop organizational and societal resilience capacity.

As for beliefs about the future, our results demonstrated a reasonably strong belief that DT, as well as clinical protocols for care, will be increasingly used in the future, while the staff in all countries has low expectations about the possibility to recruit new staff, and the overall image of health care staff in the media was also low. Overall, differences across countries were demonstrated, with staff in Germany having the lowest expectations and Italy the highest. Given the fact that Italy was hit very badly by the first wave early 2020, leading to an immense care staff burden, this result is surprising. Instead, we would have assumed that the staff in Italy would still be very affected by the pandemic at the time of our survey. It can be discussed if demographic and social factors could explain this difference among countries. Additionally, the staff in all countries did not perceive that, in the future, there will be a stronger collaboration between professionals across organizations. This is surprising since it has previously been demonstrated the there is a need for new learning in collaboration with others [56]. The COVID-19 pandemic is a widespread universal crisis, which affects the most important human value, health, and therefore, it is very sensitively perceived by the society [26]. The effect of the pandemic causes societal respect of all staff groups and the need for professional crisis management, collaboration, and organizational support at all levels: transnational, national, organizational, and individual. As demonstrated by our results, the pandemic created the need for new or adapted practices without pre-existing, evidence-based training. Thus, the need for learning new knowledge and skills in, and between, organizations and professions should be seen as a prerequisite to, and a potential for, developing health and care services for the future [56].

### Strenghts and Limitations

Data for this study were collected between 7 October 2020 and 17 December 2010, which aligned with the start of the second wave of COVID-19 in Sweden, Italy, and Germany but the middle of it for the UK. Despite the challenges faced by the societies and the participants’ considerable workload they took their time to contribute to the study. In fact, a strength of this study is the high number of survey responses from all countries. Moreover, we collected data from four countries with different health care and services systems, each of them also differently affected by the pandemic. Thus, an overview of the situation from a European perspective could be gained. However, the fact that the second wave hit the countries at different times may have affected the results since staff had different experiences of the effect of the pandemic on their work situation and the challenges faced. Additional limitations include the brief nature of the questionnaire, which was designed to be quick and easy for care staff to fill in. However, due to this, omitted questions related to contextual factors, such as COVID-19 outbreaks, within work settings, staffing levels, prior staff training and knowledge, practices, or the level of clinical skills and DT use prior to the pandemic. The questionnaire was created in English and then translated into Swedish, Italian, and German. Although translations were accurate, terms used in each country vary and can have different meanings, as some items did not fit every context. For example, terms such as ‘nursing assistant’ are widely used in Swedish care homes but not in UK care homes. The sampling strategy is likely to have affected the final sample through uptake in different countries being uneven across the different modes of survey dissemination (social media, newsletters, professional networks). Different sample sizes and distributions across professional groups and countries make some sub-groups small, and thus, our findings should be interpreted with caution. The fact that we did not ask the respondents about their previous knowledge can also be considered a limitation of the study.

## 5. Conclusions

From our study we can conclude that despite differences between countries, it seems that during the COVID-19 pandemic home care and care home staff in Europe were facing several and different challenges. Learning of new knowledge and skills in different care practice areas was achieved in all countries and professionals, and beliefs about the future were found. The care staff’s experience, reflected by the responses, should be seen as a potential to develop health and social care for the future. In this context, our study contributes to the understanding of knowledge acquisition among social and care staff in different European countries during very challenging times for the society. Further research could examine the mechanisms underlying these swift and successful practice changes, which would be useful to drive future intervention implementation and practice changes in care settings.

## Figures and Tables

**Table 1:** Learning of new knowledge and skills related to crisis management.

	Sweden		Italy		Germany		UK		Total		*p*
	*n*	M (SD)	*n*	M (SD)	*n*	M (SD)	*n*	M (SD)	*n*	M (SD)	
I have learned new knowledge and skills related to crisis management											
Manager/coordinator	16	4.19 (0.66)	24	4.33 (0.56)	31	4.32 (0.83)	65	4.02 (0.94)	136	4.16 (0.84)	0.246
Registered nurse	32	3.53 (0.92)	11	3.27 (1.49)	69	3.17 (1.28)	13	3.62 (1.19)	125	3.32 (1.21)	0.431
Nursing assistant	110	3.82 (1.00)	50	4.00 (1.12)	11	2.91 (1.38)	13	3.00 (1.22)	184	3.64 (1.11)	**0.002**
Other care staff	34	3.82 (1.00)	14	3.57 (1.28)	7	3.14 (1.35)	68	3.51 (1.25)	123	3.59 (1.19)	0.470
Total	195	3.69 (0.97)	100	3.94 (1.12)	118	3.45 (1.29)	159	3.69 (1.16)	572	3.68 (1.13)	**0.016**

*n* = number; M = Mean; SD = Standard Deviation. Significant values in bold (*p* ≤ 0.05).

**Table 2:** Learning of new knowledge and skills related to clinical and care standards.

	Sweden		Italy		Germany		UK		Total		*p*
	*n*	M (SD)	*n*	M (SD)	*n*	M (SD)	*n*	M (SD)	*n*	M (SD)	
Management/coordinator											
I have learned new knowledge and skills related to …											
infection control	16	4.56 (0.51)	23	4.30 (0.76)	29	4.10 (0.98)	65	4.20 (0.99)	133	4.24 (0.91)	0.406
care and service	15	3.93 (1.03)	23	4.26 (0.69)	29	3.00 (1.25)	65	3.98 (1.08)	132	3.81 (1.14)	**<0.001**
rehabilitation	15	3.53 (0.92)	21	3.33 (1.20)	23	2.22 (1.09)	63	3.30 (1.03)	122	3.13 (1.14)	**<0.001**
usage of PPE personaprotective technology	15	3.53 (0.92)	21	3.33 (1.20)	23	2.22 (1.09)	63	3.30 (1.03)	122	3.13 (1.14)	**<0.001**
Registered Nurse											
I have learned new knowledge and skills related to …											
infection control	36	4.03 (0.88)	12	3.42 (1.13)	70	3.34 (1.24)	14	3.86 (1.17)	132	3.59 (1.18)	**0.027**
care and service	36	3.61 (0.99)	12	3.67 (1.50)	66	2.80 (1.22)	13	4.00 (0.82)	127	3.24 (1.23)	**<0.001**
rehabilitation	32	3.19 (1.00)	12	3.00 (1.41)	64	2.34 (1.21)	13	3.08 (1.04)	121	2.71 (1.21)	**<0.001**
usage of PPE personaprotective technology	35	3.94 (1.00)	12	4.00 (1.21)	70	3.40 (1.22)	14	3.43 (1.09)	131	3.60 (1.17)	0.078
Nursing assistant											
I have learned new knowledge and skills related to …											
infection control	117	4.38 (0.72)	47	4.21 (0.93)	11	3.18 (1.33)	13	3.85 (1.14)	188	4.08 (0.95)	**0.007**
care and service	117	4.34 (0.80)	47	4.36 (0.79)	11	3.00 (1.26)	13	3.08 (1.04)	188	4.03 (0.98)	**<0.001**
rehabilitation	107	3.69 (1.15)	43	4.28 (0.93)	10	2.60 (1.26)	13	3.08 (1.19)	173	3.68 (1.09)	**<0.001**
usage of PPE personaprotective technology	116	4.35 (0.63)	46	4.52 (0.75)	11	3.27 (1.35)	13	3.54 (1.33)	186	4.24 (0.96)	**<0.001**
Other staff											
I have learned new knowledge and skills related to…											
infection control	37	4.38 (0.72)	14	3.36 (1.34)	6	3.50 (1.38)	73	4.16 (1.05)	130	4.11 (1.06)	**0.008**
care and service	35	4.34 (0.80)	14	3.64 (1.34)	7	2.86 (1.46)	74	3.93 (1.19)	130	3.95 (1.17)	**0.010**
rehabilitation	32	3.69 (1.15)	13	3.62 (1.12)	5	2.00 (1.00)	69	3.58 (1.16)	119	3.55 (1.18)	**0.025**
usage of PPE personaprotective technology	37	4.35 (0.63)	13	3.92 (1.12)	7	3.71 (1.25)	74	4.14 (1.15)	131	4.15 (1.03)	0.352

PPE = personal protective equipment; *n* = number; M = Mean; SD = Standard Deviation. Significant values in bold (*p* ≤ 0.05).

**Table 3:** Learning of new knowledge and skills related to use of digital technology.

	Sweden		Italy		Germany		UK		Total		*p*
	*n*	M (SD)	*n*	M (SD)	*n*	M (SD)	*n*	M (SD)	*n*	M (SD)	
Management/coordinator											
I have learned new knowledge and skills related to digital technology…											
for communication	16	4.13 (0.96)	24	3.79 (1.28)	30	3.33 (1.47)	65	3.77 (1.10)	135	3.72 (1.22)	0.174
for infection tracing	15	3.40 (1.18)	22	2.64 (1.40)	27	2.22 (1.40)	64	3.39 (1.18)	128	3.02 (1.35)	**<0.001**
to support care	16	3.63 (1.20)	20	3.05 (1.28)	26	2.00 (1.26)	65	3.65 (1.11)	127	3.21 (1.34)	**<0.001**
Registered nurse											
I have learned new knowledge and skills related to digital technology											
for communication	34	3.76 (0.82)	12	3.42 (1.31)	69	2.88 (1.52)	12	2.75 (1.22)	127	3.16 (1.37)	**0.010**
for infection tracing	26	2.92 (1.13)	12	2.83 (1.47)	65	2.26 (1.31)	12	2.25 (1.36)	115	2.47 (1.31)	0.111
to support care	31	2.94 (1.26)	12	3.33 (1.50)	67	2.03 (1.28)	12	2.75 (1.60)	122	2.46 (1.40)	**0.001**
Nursing assistant											
I have learned new knowledge and skills related to digital technology											
for communication	113	3.75 (0.98)	47	4.36 (0.85)	10	3.00 (1.63)	13	3.54 (1.33)	183	3.83 (1.06)	**<0.001**
for infection tracing	96	3.46 (1.35)	47	4.11 (1.03)	10	2.80 (1.40)	13	2.62 (1.66)	166	3.35 (1.27)	**<0.001**
to support care	105	3.43 (1.30)	47	4.19 (1.08)	9	2.78 (1.20)	13	2.62 (1.56)	174	3.49 (1.23)	**<0.001**
Other staff											
I have learned new knowledge and skills related to…											
for communication	32	3.75 (0.98)	14	3.64 (1.55)	5	2.40 (1.14)	73	3.66 (1.28)	124	3.63 (1.25)	0.161
for infection tracing	28	3.46 (1.35)	14	3.07 (1.44)	6	1.33 (0.52)	68	3.60 (1.38)	116	3.39 (1.43)	**0.002**
to support care	30	3.43 (1.30)	14	3.14 (1.23)	6	1.67 (1.03)	71	3.55 (1.34)	121	3.38 (1.36)	**0.009**

*n* = number; M = Mean; SD = Standard Deviation. Significant values in bold (*p* ≤ 0.05).

**Table 4:** Beliefs about the future, after the pandemic, by profession and country.

	Sweden		Italy		Germany		UK		Total		*p*
	*n*	M (SD)	*n*	M (SD)	*n*	M (SD)	*n*	M (SD)	*n*	M (SD)	
In the future I believe that digital technology will be more common in care services											
Manager/coordinator	16	4.50 (0.52)	24	4.58 (0.50)	31	4.32 (0.87)	65	4.38 (0.82)	136	4.42 (0.76)	0.586
Registered nurse	34	4.35 (0.65)	12	4.17 (1.11)	68	3.79 (1.04)	14	3.79 (1.12)	128	3.98 (0.99)	**0.041**
Nursing assistant	115	4.30 (0.73)	50	4.62 (0.60)	10	4.50 (0.53)	13	4.38 (0.65)	188	4.40 (0.69)	0.057
Other care staff	37	4.27 (0.80)	14	4.29 (1.07)	6	4.00 (1.55)	70	4.44 (0.83)	127	4.35 (0.89)	0.563
Total	205	4.32 (0.72)	101	4.51 (0.74)	115	4.01 (1.02)	163	4.36 (0.85)	584	4.30 (0.84)	**<0.001**
In the future I believe that application of clinical protocols for infection control will become more common											
Manager/coordinator	15	4.40 (0.63)	24	4.63 (0.49)	29	3.62 (0.90)	65	4.40 (0.61)	133	4.27 (0.75)	**<0.001**
Registered nurse	35	4.37 (0.65)	12	4.17 (1.11)	65	3.40 (1.13)	14	4.36 (0.50)	126	3.85 (1.06)	**<0.001**
Nursing assistant	114	4.38 (0.73)	49	4.55 (0.71)	10	4.00 (0.47)	13	4.23 (0.60)	186	4.39 (0.71)	0.103
Other care staff	36	4.33 (0.27)	12	4.33 (1.15)	5	3.60 (1.52)	72	4.44 (0.65)	125	4.37 (0.78)	0.128
Total	203	4.37 (0.70)	98	4.5 (0.79)	109	3.52 (1.05)	165	4.40 (0.61)	575	4.24 (0.85)	**<0.001**
In the future I believe that it will be easier to attract new colleagues											
Manager/coordinator	15	2.87 (0.92)	21	2.38 (0.92)	30	1.40 (0.86)	65	2.75 (1.31)	131	2.40 (1.24)	**<0.001**
Registered nurse	34	2.44 (0.89)	12	2.42 (1.24)	67	1.93 (1.11)	14	2.64 (1.45)	127	2.19 (1.13)	**0.043**
Nursing assistant	112	2.59 (1.11)	48	3.63 (1.14)	9	2.11 (0.93)	13	2.31 (1.18)	182	2.82 (1.21)	**<0.001**
Other care staff	32	3.13 (1.31)	13	3.23 (1.36)	6	1.00 (0.00)	68	3.07 (1.33)	119	3.00 (1.37)	**0.003**
Total	196	2.66 (1.11)	95	3.13 (1.26)	112	1.75 (1.04)	161	2.85 (1.33)	564	2.61 (1.27)	**<0.001**
In the future I believe that there will be a stronger collaboration between professionals across organizations											
Manager/coordinator	15	3.47 (0.74)	24	3.21 (0.98)	31	2.19 (1.28)	64	3.20 (1.22)	134	3.00 (1.23)	**<0.001**
Registered nurse	34	3.18 (0.94)	12	2.75 (1.22)	65	2.28 (1.17)	14	3.36 (0.74)	125	2.69 (1.15)	**<0.001**
Nursing assistant	102	3.30 (1.02)	48	4.10 (0.88)	10	2.60 (0.97)	13	3.08 (1.32)	173	3.47 (1.09)	**<0.001**
Other care staff	34	3.59 (1.02)	13	3.77 (1.09)	4	2.25 (0.96)	73	3.30 (1.31)	124	3.40 (1.22)	0.110
Total	188	3.35 (0.98)	98	3.65 (1.09)	110	2.28 (1.17)	165	3.25 (1.23)	561	3.17 (1.2)	**<0.001**
In the future I believe that the public image of care staff will be better in the media											
Manager/coordinator	15	4.00 (0.93)	24	3.58 (1.06)	30	2.30 (1.15)	64	3.08 (1.35)	133	3.10 (1.31)	**<0.001**
Registered nurse	34	3.26 (0.96)	12	2.50 (1.31)	68	2.15 (1.18)	14	3.07 (1.07)	128	2.58 (1.22)	**<0.001**
Nursing assistant	112	3.06 (1.23)	49	3.96 (1.06)	11	2.27 (1.10)	13	2.54 (1.33)	185	3.22 (1.28)	**<0.001**
Other care staff	34	3.50 (1.08)	13	3.15 (1.21)	7	2.00 (1.00)	71	3.27 (1.32)	125	3.25 (1.26)	**0.038**
Total	198	3.24 (1.17)	99	3.57 (1.21)	116	2.19 (1.14)	163	3.12 (1.31)	576	3.05 (1.29)	**<0.001**

*n* = number; M = Mean; SD = Standard Deviation. Significant values in bold (*p* ≤ 0.05).

**Table 5:** Associations between new knowledge and learning and beliefs about the future.

	Model 1 ^1^		Model 2 ^2^	
Country (ref: Germany)			Coefficient	*p*
Sweden	0.37 ± 0.27	0.167	0.18 ± 0.33	0.588
Italy	0.94 ± 0.30	**0.002**	0.13 ± 0.36	0.714
UK	0.42 ± 0.28	0.132	−0.04 ± 0.34	0.898
Age	−0.01 ± 0.01	0.172	−0.01 ± 0.01	0.168
Gender (ref: male)				
Female	−0.07 ± 0.28	0.789	0.01 ± 0.32	0.985
Non-binary	0.04 ± 1.64	0.979	−1.19 ± 1.74	0.495
Prefer not to say	1.23 ± 1.25	0.324	0.85 ± 1.39	0.539
Profession (ref: management)				
Registered nurse	−0.98 ± 0.27	**<0.001**	−0.61 ± 0.32	0.058
Nursing assistant	−0.29 ± 0.26	0.255	−0.17 ± 0.30	0.572
Other profession	−0.12 ± 0.26	0.649	0.23 ± 0.31	0.449
During the COVID-19 pandemic I have learned new knowledge and skills related to crisis management (ref: strongly disagree)				
Mostly disagree			−0.21 ± 0.47	0.653
Agree or disagree			−0.23 ± 0.41	0.575
Mostly agree			0.05 ± 0.41	0.894
Strongly agree			1.19 ± 0.46	**0.011**
During the COVID-19 pandemic I have learned new knowledge and skills related to usage of digital technology to support care (ref: strongly disagree)				
Mostly disagree			0.54 ± 0.34	0.111
Agree or disagree			0.37 ± 0.31	0.24
Mostly agree			1.26 ± 0.33	**<0.001**
Strongly agree			2.29 ± 0.42	**<0.001**

Significant values in bold (*p* ≤ 0.05). ^1^ Model 1 includes individual characteristics. ^2^ Model 2 includes individual characteristics and new knowledge and learning.

## Data Availability

Data is available on reasonable request.

## Supplementary Materials

The following supporting information can be downloaded at: https://www.mdpi.com/article/10.3390/healthcare10020306/s1, COVID-19 Professionals experiences in care homes and home care.
