# Supplementary material for: Care Home and Home Care Staff’s Learning during the COVID-19 Pandemic and Beliefs about Subsequent Changes in the Future: A Survey Study in Sweden, Italy, Germany and the United Kingdom
Source: Healthcare (Basel). 2022 Feb 5;10(2):306. doi: 10.3390/healthcare10020306 (PMC8872186; doi:10.3390/healthcare10020306)
Supplement: Supplementary file 1 [file healthcare-10-00306-s001.zip › healthcare-1566063-supplementary.pdf]

COVID-19 Professonals experiences in care homes and home care ENG

COVID-19 IN CARE HOMES AND HOME CARE -
PROFESSIONALS EXPERIENCES, ORGANIZATIONAL SUPPORT AND STRATEGIES FOR LEARNING AND NEW KNOWLEDGE

Do you work in a care home or in home care? Does COVID-19 cause challenges in your working situation? What learning experiences have you had in your work during the crisis? We would very much like to ask you a few questions in order to learn how what could help you, now and in the future.

We are researchers at Lund University and Malmö University collaborating with researchers from the United Kingdom (University of East Anglia), Germany, and Italy.

This study has been reviewed by the Research Ethics Committee, Faculty of Medicine and Health, University of East Anglia. We do not anticipate any risk to you of taking part, but should the issues raise difficult feelings you could contact the Samaritans Wellbeing Support Line for health and social care staff tel:0800 069 6222. If you need to make a complaint about the study contact Professor Sally Hardy, Head of School, School of Health Sciences, University of East Anglia, Norwich, NR4 7TJ. Data will be stored securely on a database at Lund University, in compliance with GDPR regulations. Anonymised data only will be stored.

You do not have to take part in this survey. The survey is completely anonymous and your

answers cannot be traced back to you. If you want to be informed about the results, you are most welcome to contact us. By replying to the questions you give us permission to save and collate your answers into a file with responses from others nursing home staff in the United Kingdom, Germany, Italy, and Sweden. If you changed your mind about taking part it would not be possible to take your survey answers out of the data as only anonymised data is stored. Please share the survey also with your colleagues. If you want to know more about the study contact Anne Killett, a.killett@uea.ac.uk.

PART 1 - CHARACTERISTICS OF THE RESPONDENTS

1) What is your age (in years)?

____

2) How would you describe your gender identity...

◯ Male
◯ Female
◯ Non-binary/third gender
◯ Prefer not to say

3) Professional role

◯ Manager/coordinator
◯ Registered nurse
◯ Nurse assistant
◯ Other care professional

4) I work in...

◯ Public organization
◯ Private organization
◯ Non Govermental Organization

5) I work in a care home or in home care

◯ Care home
◯ Home care

6) Region/county

◯ England
◯ Scotland
◯ Wales
◯ Northern Ireland

PART 2 - YOUR WORK EXPERIENCE DURING THE PANDEMIC

How would you rate the impact that the COVID-19 pandemic has had on the stress and anxiety that you have experienced during the last month, on a scale from 1 (no impact) to 5 (a very strong impact)?

		1	2	3	4	5
7)	Stress and anxiety	◯	◯	◯	◯	◯

PART 3 - PERCEIVED INSTITUTIONAL AND ORGANIZATIONAL SUPPORT DURING THE PANDEMIC

3.1 Please rate your level of agreement/disagreement with the following statements.

With respect to the organization where I work...

		Strongly agree	Mostly agree	Nor agree or disagree	Mostly disagree	Strongly disagree	Don't Know/No Opinion
8)							

	I have received clear guidelines on how to behave at work	○	○	○	○	○	○
9)	I have received an adequate provision of personal protection equipment	○	○	○	○	○	○
10)	I have received an adequate training on procedures for care and protection	○	○	○	○	○	○
11)	My management supported me adequately	○	○	○	○	○	○
12)	My voice has been listened to by my organization	○	○	○	○	○	○
13)	I have been able to take the holiday leave I am entitled to this year	○	○	○	○	○	○

3.2 Please express your level of agreement/disagreement with the following statements.

With respect to the regional context where my organization is located...

		Strongly agree	Mostly agree	Nor agree or disagree	Mostly disagree	Strongly disagree	Don't Know/No Opinion
14)	My organization has received clear guidelines on how to behave during the pandemic	○	○	○	○	○	○
15)	The community where my organization is located has strongly supported us	○	○	○	○	○	○
16)	The families of the residents have been supportive and collaborative	○	○	○	○	○	○

PART 4 - LEARNING AND NEW STRATEGIES DURING THE PANDEMIC

Please express your level of agreement/disagreement with the following statements.

During the COVID-19 pandemic I have learned new knowledge and skills related to...

		Strongly agree	Mostly agree	Nor agree or disagree	Mostly disagree	Strongly disagree	Don't Know/No Opinion
17)	Crisis management	○	○	○	○	○	○
18)	Infection control	○	○	○	○	○	○
19)							

	Strongly agree	Mostly agree	Nor agree or disagree	Mostly disagree	Strongly disagree	Don't Know/No Opinion
Care and service	○	○	○	○	○	○
20) Rehabilitation	○	○	○	○	○	○
21) Usage of personal protection equipment	○	○	○	○	○	○
22) Usage of digital technology for communication	○	○	○	○	○	○
23) Usage of digital technology for infection tracing	○	○	○	○	○	○
24) Usage of digital technology to support care	○	○	○	○	○	○

PART 5 - PERSPECTIVE FOR THE FUTURE

Please express your level of agreement/disagreement with the following statements.

In the future I believe that...

	Strongly agree	Mostly agree	Nor agree or disagree	Mostly disagree	Strongly disagree	Don't Know/No Opinion
25) Digital technology will be more common in care and services	○	○	○	○	○	○
26) Application of clinical protocols for infection control will become more common	○	○	○	○	○	○
27) It will be easier to attract new colleagues	○	○	○	○	○	○
28) There will be a stronger collaboration between professionals across organizations	○	○	○	○	○	○
29) The public image of care staff will be better in the media	○	○	○	○	○	○

Thank you for your participation - Your contribution is valuable. If you want to take part of the results, please contact: carlos.chiatti@med.lu.se
